# Safety of High-Dose Intravenous Iron in Hemodialysis Patients: Results from the National Health Insurance Service (2019–2020) in South Korea

**DOI:** 10.3390/jcm14010063

**Published:** 2024-12-26

**Authors:** AJin Cho, Yoonjong Bae, Mina Kim, Do Hyoung Kim, Young-Ki Lee, Hayne Cho Park

**Affiliations:** 1 Department of Internal Medicine, Kangnam Sacred Heart Hospital, Hallym University College of Medicine, Singil-ro, Yeongdeungpo-gu, Seoul 07441, Republic of Korea; dhkim6489@hallym.or.kr (D.H.K.); km2071@hallym.or.kr (Y.-K.L.);; 2 Kidney Research Institute, Hallym University, Seoul 07441, Republic of Korea; 3 Department of Data Science, Hanmi Pharm. Co., Ltd., Seoul 05545, Republic of Korea

**Keywords:** intravenous, iron, hemodialysis, infection, mortality, hospitalization

## Abstract

**Background**: Intravenous (IV) iron administration is used widely for treating anemia in hemodialysis (HD) patients. In this study, we investigated the safety of IV iron therapy in this population. **Methods**: This study analyzed claims data from the National Health Insurance Service (NHIS) and included patients with end-stage renal disease who were receiving HD for more than 3 months as of 1 January 2019. Monthly doses of IV iron were measured for these patients from 1 January to 30 June 2019. Patients were classified into a high- or low-dose group based on the cutoff of a monthly dose of 300 mg of iron sucrose. Study outcomes were infection-related hospitalization, cardiovascular events, and all-cause mortality and hospitalization that occurred from 1 July 2019 to 31 December 2020. **Results**: Among 33,527 HD patients, 13,609 (40.6%) and 363 (1.1%) patients were administered IV iron at doses of 1–299 mg/month and ≥300 mg/month, respectively. The mean age was 63 years, and 60.4% were men. Compared with the low-dose group, the high-dose group was younger, had higher percentages of men and medical aid recipients from the NHIS, and had higher prevalence rates of diabetes and hypertension. The rates of infection-related hospitalization, cardiovascular events, and all-cause hospitalization and mortality were not significantly higher in the high-dose than in the low-dose group. Compared with the 1–100 mg IV iron sucrose dose, higher doses were not associated with an increased risk of outcome events. **Conclusions**: High-dose IV iron administration did not increase rates of mortality or morbidity in HD patients.

## 1. Introduction

Anemia is common in hemodialysis (HD) patients who are typically iron deficient because of iron malabsorption and blood loss during HD [1]. Intravenous (IV) iron has become a standard treatment in the management of anemia and is used to reduce the need for erythropoiesis-stimulating agents (ESAs) to save costs and to alleviate the potential risks associated with higher doses of ESAs [2,3,4]. Clinical studies have noted the side effects of high-dose ESAs, including elevated hemoglobin levels, and that these are associated with cardiovascular complications [3,5,6]. In 2012, the Kidney Disease Improving Global Outcomes (KDIGO) guidelines for anemia management suggested wider use of iron agents for the treatment of anemia in patients undergoing dialysis or with nondialysis chronic kidney disease (CKD) [7]. Oral iron preparations are cost-effective and convenient to administer. However, increased hepcidin concentration in CKD patients may limit iron availability in the intestine and in macrophages, which may lead to poor absorption of iron [8]. For this reason, IV iron is used to increase the hemoglobin level while replenishing iron with minimal use of ESAs.

The safety of IV iron in patients undergoing HD is unclear. The KDIGO guideline recommendations express concerns about the risks of IV iron administration [7]. IV iron is known to have negative in vitro effects on the immune system and to have variable effects on infection risk [9,10,11]. Free iron is a powerful oxidant that leads to the formation of reactive oxygen species. Free radicals can turn into lipid radicals, which can lead to endothelial dysfunction and atherosclerosis [12,13,14]. Previous observational studies have reported conflicting findings regarding an association between IV iron and mortality. In the Dialysis Outcomes and Practice Patterns Study (DOPPS) of more than 30,000 patients undergoing HD, patients receiving IV iron at a dose of ≥300 mg/month had a significantly higher risk of mortality and hospitalization compared with those on lower doses [15]. Conversely, the Proactive IV Iron Therapy in Haemodialysis Patients (PIVOTAL) study reported a trend toward decreased mortality in those receiving IV iron at a dose of >400 mg/month. In the safety analysis, the mortality rate in high- and low-dose groups was similar during the mean 2.1-year follow-up period [16].

Intravenous iron utilization strategies for patients undergoing dialysis vary widely between countries, and ongoing concerns remain regarding the safety of this practice. In Korea, IV iron is used according to insurance standards under the National Health Insurance Service (NHIS). However, there is a lack of consensus about the optimal dose that does not induce side effects. In this study, we examined the NHIS claims data to investigate the safety of IV iron in patients undergoing HD.

## 2. Methods

### 2.1. Data Source

The NHIS in Korea is a single-payer system to which 98% of citizens belong. Under this system, medical providers request reimbursement from the NHIS. We conducted a retrospective cohort study based on the NHIS claims data. The study was conducted in accordance with the Declaration of Helsinki. The database was fully anonymized, and the requirement of informed consent was waived by the ethics committee of the Institutional Review Board of Hallym University Kangnam Sacred Heart Hospital, Seoul, South Korea (IRB No. HKS 2024-01-004).

### 2.2. Study Population and Design

**Figure 1:**
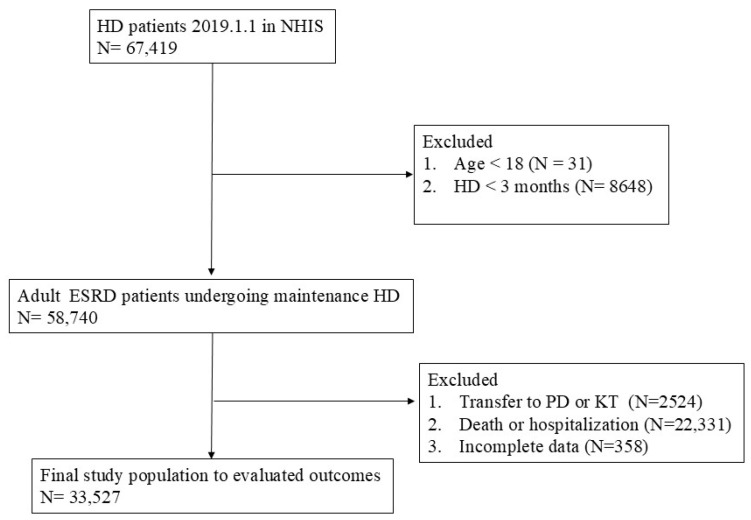
Flow diagram of the study population.

The target patients were patients with end-stage renal disease (ESRD) who were receiving HD for >3 months as of 1 January 2019 (index date). To select patients for the study, we first selected those with CKD using the International Classification of Diseases codes (tenth revision, ICD-10) N18.0–18.6 and 18.9. Patients with ESRD were then selected using the special exemption codes V001 (HD), V003 (peritoneal dialysis, PD), and V005 (kidney transplant, KT) provided by the Korean government. HD patients were classified based on these special exemption codes and procedure codes (HD: O7020, O7021, and O9991; PD: O707; and KT: R3280) from the NHIS claims. Monthly doses of IV iron were examined for these patients from 1 January to 30 June 2019. A total of 67,419 HD patients were first identified, and those whose dialysis modality was changed to PD or who received a KT were excluded. HD patients who died or were hospitalized from January to June 2019 were also excluded from the study (Figure 1).

The included patients were classified into a high- or low-dose group based on the monthly iron sucrose dosage administered intravenously from 1 January to 30 June 2019. We examined the total IV iron doses per month and used the average monthly dose over a six-month period to define high- and low-dose groups [17]. Patients in the high-dose group received a monthly dose of ≥300 mg of IV iron sucrose. Patients in the low-dose group received a monthly dose of 1–299 mg of IV iron sucrose. In Korea, IV iron is administered to HD patients according to insurance standards. In these standards, this treatment is approved by insurance and administered when the serum hemoglobin level is less than 11 g/dL, the serum ferritin level is less than 200 ng/mL, or the transferrin saturation is less than 20%. Patients who do not meet these criteria are prescribed oral iron supplements or do not take iron.

### 2.3. Outcomes

The study outcomes were infection-related hospitalization, cardiovascular events, and all-cause mortality and hospitalization that occurred from 1 July 2019 to 31 December 2020. We used ICD-10 and procedure codes for identifying outcome events (Supplementary File S1).

### 2.4. Covariates

Demographic data (age and sex), medical aid status from the NHIS, and data regarding comorbid conditions (diabetes, hypertension, heart failure, ischemic heart disease, cerebrovascular disease, liver cirrhosis and failure, chronic obstructive lung disease, and cancer) were collected. Comorbidities were determined using the corresponding ICD-10 codes (Supplementary File S1) more than once during hospitalization and more than twice in outpatient settings by referring to claims data from 1 year before the index date (1 January 2019).

### 2.5. Statistical Analysis

Summary statistics are provided as numbers and percentages or as mean values with standard deviations. The Kolmogorov–Smirnov test was used to evaluate the normal distribution. Chi-square analysis was used to compare categorical variables, and analysis of variance was used to compare continuous variables between the groups. Between-group post hoc tests were performed using Bonferroni post hoc tests. Cox proportional hazard analysis was performed to identify the risk of outcomes according to IV iron dose. The dependent variables were infection-related hospitalizations, cardiovascular events, and all-cause mortality and hospitalizations, and IV iron dose was the independent variable. Multivariable analyses were adjusted for age, sex, medical aid beneficiary status, and comorbid conditions (diabetes, hypertension, heart failure, ischemic heart disease, cerebrovascular disease, chronic obstructive pulmonary disease (COPD), liver cirrhosis, and cancer). In the survival analysis of outcomes, high- and low-dose groups were matched in a 1:3 ratio using propensity scores to minimize confounding factors that could affect the results. Propensity score matching was performed based on age, sex, medical aid status, and comorbid conditions. For the propensity score matching process, we used the greedy matching method, and the caliper was set at 0.25. All *p*-values were two-sided, and *p* < 0.05 was considered significant. Statistical analyses were performed using SAS version 9.4 (SAS Institute, Cary, NC, USA).

## 3. Results

Among the 33,527 HD patients, 13,609 (40.6%) and 363 (1.1%) patients were administered IV iron at 1–299 mg/month and ≥300 mg/month, respectively. More than half (19,555, or 58.3%) of the patients did not take iron supplements (the no-IV iron group) or took oral iron. The mean age of the patients was 63 years; 60.4% were men, and 24.5% were medical aid recipients. Compared with the low-dose group, the high-dose group was younger, had higher percentages of men and medical aid recipients, and had higher prevalence rates of diabetes, hypertension, and COPD (Table 1).

The hazard ratios of the outcome events are presented in Table 2. The risk rates of infection-related hospitalization (adjusted hazard ratio (aHR), 1.098; 95% confidence interval (CI), 0.856–1.408) and cardiovascular events (aHR, 0.919; 95% CI, 0.733–1.153) were not significantly higher in the high-dose than in the low-dose IV iron group. All-cause hospitalization and mortality rates also did not differ between these two groups. The risk rates of infection-related hospitalization and cardiovascular events did not differ between the no-IV iron group and the low-dose IV iron group. However, all-cause hospitalization and mortality risk rates were lower in the no-IV iron group than in the low-dose IV iron group.

Next, we analyzed the risk of outcomes with reference to the group of patients who received a monthly dose of 1–100 mg of IV iron (Table 3). Compared with the 1–100 mg iron group, the risk of outcome events was not increased in the higher-dose IV iron and no-IV groups. The baseline characteristics of the high- and low-dose IV iron groups after propensity score matching are presented in Supplementary File S2, which shows that the clinical variables were well-balanced between the two groups. After propensity score matching, the risk of the outcome events did not differ between the high- and low-dose IV iron groups (Table 4).

Subgroup analyses between the high- and low-dose IV iron groups are shown in Table 5. The two groups did not differ on the variables of age, sex, receipt of medical aid, diabetes, ischemic heart disease, and heart failure.

## 4. Discussion

In this study, the use of high-dose IV iron was not associated with an increased risk of infection, cardiovascular events, hospitalizations, or mortality compared with low-dose IV iron in HD patients. After the Trial to Reduce Cardiovascular Events with Aranesp Therapy (TREAT) study was published, coadministration of iron and ESAs has led to increased interest in using low doses of ESAs [6]. IV iron supplements administered are used widely to treat anemia in HD patients and have been shown to be effective in treating anemia [15,16,18]. However, the safety of IV iron treatment for HD patients is less certain. In Korea, IV iron is used according to insurance standards, and oral iron is discontinued during the period of IV iron use. However, IV iron is being administered without clear guidelines for the treatment of anemia in HD patients, and the safe dosage has not been determined.

Iron supplementation may increase the risk of infection by impairing neutrophil and T-cell function and promoting microbial growth [19,20,21]. This increased risk is particularly important because infections are a significant cause of mortality and morbidity in HD patients. A meta-analysis of 72 randomized controlled trials (RCTs) that included 10,605 people to evaluate the efficacy and safety of IV iron showed that this treatment was associated with an increase in hemoglobin concentration and a 26% reduced risk of needing red blood cell transfusion [22]. However, IV iron was associated with a 33% higher risk of any infection compared with oral or no iron supplementation. The results of that meta-analysis remained similar when only high-quality trials were analyzed. A prospective study of 985 HD patients failed to find a significant association between IV iron administration and the risk of bacteremia. However, there was a slightly increased risk of bacteremia in patients receiving high-frequency, high-dose IV iron [23]. The DOPPS study results showed a trend toward increased infection-related mortality in patients receiving IV iron at doses >300 mg/month, but this was not statistically significant [17]. A systematic review and meta-analysis of 7 RCTs and 15 observational studies of 140,000 participants undergoing HD found no evidence of increased risk of infection, cardiovascular events, hospitalizations, or mortality with the higher dose compared with lower doses of IV iron. For the study, the high-dose iron group was defined as 200 mg and 400 mg or more per month in observational studies and RCTs, respectively [24]. Previous studies have shown that IV iron use does not increase the risk of infection in HD patients and that higher doses do not increase the risk of infection. The results of this study support these conclusions.

The results to date have been inconsistent regarding the safety of IV iron in HD patients. Results from experimental studies of iron supplementation on infection risk do not always apply to clinical settings [20,21], possibly because of the short-term use (1 to 6 months) of iron in previous studies [24]. Our study also set the 6-month IV iron dose as the cutoff between the high- and low-dose groups. This time limit was chosen because of the high mortality and morbidity of HD patients; that is, a longer period of iron use may have captured the increasing incidence of adverse reactions to other causes, which would have made it difficult to determine the response to IV iron supplementation. These issues may also apply in studies with a long-term follow-up of the safety of IV iron. It is possible that the physical condition was more severe in patients receiving IV iron than in those receiving oral iron or no iron, which may have affected the interpretation of our results. In this study, the high-dose IV iron group was younger but included a higher percentage of patients with comorbid conditions compared with the low-dose group. However, the risk of outcome events did not differ after propensity score matching.

Patients with CKD are in a pro-oxidative state, which leads to accelerated atherosclerosis and subsequent excessive cardiovascular death [25]. A central role in the development of atherosclerosis in CKD is endothelial dysfunction mediated by oxidative stress. IV iron has been shown to be an important contributor to oxidative stress in CKD [26]. Free iron is a potent oxidant, forming free radicals that can rise to lipid radicals and cause endothelial dysfunction and atheroma formation [27]. However, these conclusions should be carefully considered in the context of the iron formulation used. Several studies showed that the effects of different IV iron complexes on oxidative stress and inflammation varied depending on the iron complex [28,29,30]. Ferric gluconate, which is known to release large amounts of labile iron, showed significant signs of oxidative stress and inflammation, whereas iron sucrose and ferric carboxymaltose did not when compared to saline controls. Similarly, a study comparing an iron sucrose originator product to six iron sucrose analogs showed significant differences in the markers of oxidative stress and inflammatory response [30]. Epidemiologic data on the association between IV iron and cardiovascular disease are inconsistent [17,31,32]. Despite the pathogenicity hypothesis, evidence relating to IV iron therapy, oxidative stress, and cardiovascular disease remains limited.

Meanwhile, IV iron supplementation has beneficial effects in patients with heart failure, suggesting that iron acts directly on heart function [33]. The possible mechanism may involve the important role of iron in cellular oxygen storage and cell metabolism in cardiomyocytes with a high energy demand [34]. Iron replenishment may also benefit skeletal muscle function and contribute to the improvement in quality of life and general functional capacity after iron therapy in patients with chronic heart failure. RCTs have shown that IV iron improves exercise capacity and quality of life [35,36]. In a meta-analysis of 12 RCTs of patients with heart failure, IV iron administration reduced the hospitalization rate for heart failure, but the association with cardiovascular death was not significant [33]. The PIVOTAL trial showed that high-dose IV iron resulted in a significantly lower risk of major nonfatal cardiovascular events compared with a low-dose regimen among HD patients [16]. In that study, high-dose IV iron administration had a positive effect on the cardiovascular system by reducing the use of ESAs and maintaining a high blood hemoglobin concentration. The beneficial effects of iron on the cardiovascular system may have helped to counteract the risk of high-dose IV iron in our study.

This study has several limitations. First, this observational study cannot establish causality, and it remains susceptible to the possibility of residual confounding. Second, because this study included Korean HD patients, the ability to generalize the findings to other populations is limited. Third, we could not obtain data for the serum levels of ferritin and hemoglobin and the doses of erythropoietin. However, it is known from previous research that IV iron administration increases hemoglobin concentration and reduces the dose of erythropoietin needed [4,16]. We also did not have data on dialysis treatment details, such as frequency, duration, or adequacy of dialysis sessions, which are important factors that can affect patient outcomes and interact with intravenous iron therapy. Fourth, the number of patients in the subgroups separated by IV iron dose was uneven. However, many clinicians are concerned about the safety of high-dose IV iron, which may explain why the number of patients in each group was uneven. This study is an observational study, and to overcome this issue, we analyzed survival analyses between the high-dose and low-dose groups after propensity score matching. Fifth, the observation period was short, and larger and higher-quality RCTs will be needed to confirm these findings. Sixth, we did not monitor changes in comorbidity status during the study but adjusted for baseline comorbidities in multivariate analyses. Finally, we excluded patients who died or were hospitalized between January and June 2019, which may have contributed to the survival bias.

In conclusion, this study found no evidence of excess infections, cardiovascular events, hospitalization, or death associated with high-dose IV iron supplementation. Given these results, the safety of IV iron administration cannot be assured in HD patients. However, 6 months of administration did not seem to increase mortality or morbidity in these HD patients. Considering that most patients do not receive IV iron therapy continuously but take a rest period while their ferritin level is monitored, the results of this study may help alleviate nephrologists’ concerns about the potential side effects of IV iron therapy.

## Figures and Tables

**Table 1:** Baseline characteristics of the study population by intravenous iron dose.

	Total	No-IV Iron	Low-Dose Iron 1–299, mg/month	High-Dose Iron ≥300, mg/month	*p*-Value
	(n = 33,527)	(n = 19,555)	(n = 13,609)	(n = 363)	
Age (years)	63.17 ± 12.80	63.35 ± 12.69	63.01 ± 12.95	59.63 ± 13.05	<0.001
18–64	17,755 (52.96)	10,275 (52.54)	7249 (53.27)	231 (63.64)	<0.001
≥65	15,772 (47.04)	9280 (47.46)	6360 (46.73)	132 (36.36)	<0.001
Sex (male)	20,259 (60.43)	11,991 (61.32)	8016 (58.90)	252 (69.42)	<0.001
Sex (female)	13,268 (39.57)	7564 (38.68)	5593 (41.10)	111 (30.58)	<0.001
Medical aid recipient	8214 (24.50)	5009 (25.62)	3115 (22.89)	90 (24.79)	<0.001
Comorbid conditions					
Diabetes	19,684 (58.71)	11,436 (58.48)	8013 (58.88)	235 (64.74)	0.052
Hypertension	25,144 (75.00)	14,458 (73.94)	10,388 (76.33)	298 (82.09)	<0.001
Heart failure	8388 (25.02)	4962 (25.36)	3329 (24.46)	97 (26.72)	0.123
Ischemic heart disease	10,760 (32.09)	6132 (31.36)	4505 (33.10)	123 (33.88)	0.003
CVD	4406 (13.14)	2620 (13.40)	1744 (12.82)	42 (11.57)	0.193
COPD	9639 (28.75)	5526 (28.26)	3999 (29.39)	114 (31.41)	0.043
Liver cirrhosis and failure	988 (2.95)	567 (2.90)	409 (3.01)	12 (3.31)	0.751
Cancer	2266 (6.76)	1293 (6.61)	939 (6.90)	34 (9.37)	0.083

Data are number (percent) and mean (standard deviation). Abbreviations: CVD, cerebrovascular disease; COPD, chronic obstructive lung disease.

**Table 2:** Association between intravenous iron dose and study outcomes.

	Number of Events	Incidence (n/100,000 Persons)	Unadjusted HR (95% CI)	Adjusted HR (95% CI)
Infection-related hospitalization				
0, mg/month	3199	16,359	0.986 (0.934–1.041)	0.978 (0.927–1.033)
1–299, mg/month	2246	16,504	1 (ref)	1 (ref)
≥300, mg/month	64	17,631	1.068 (0.833–1.370)	1.098 (0.856–1.408)
Cardiovascular events				
0, mg/month	4447	23,036	0.982 (0.938–1.028)	0.976 (0.933–1.022)
1–299, mg/month	3135	21,212	1 (ref)	1 (ref)
≥300, mg/month	77	22,741	0.914 (0.729–1.146)	0.919 (0.733–1.153)
All-cause mortality				
0, mg/month	1520	7773	0.925 (0.857–0.999)	0.908 (0.840–0.980)
1–299, mg/month	1138	8362	1 (ref)	1 (ref)
≥300, mg/month	29	7989	0.955 (0.660–1.380)	1.024 (0.708–1.481)
All-cause hospitalization				
0, mg/month	10,843	55,449	0.956 (0.928–0.984)	0.954 (0.927–0.982)
1–299, mg/month	7756	56,992	1 (ref)	1 (ref)
≥300, mg/month	215	59,229	1.068 (0.932–1.223)	1.074 (0.937–1.230)

**Table 3:** Hazard ratio (95% confidence interval) of outcomes by intravenous iron dose.

Iron Dose	Number at Risk	Infection-Related Hospitalization	Cardiovascular Events	All-Cause Mortality	All-Cause Hospitalization
0	19,555	0.989 (0.919–1.066)	1.015 (0.949–1.087)	0.936 (0.845–1.036)	0.951 (0.9–1.006)
1–100, mg/month	5981	1 (ref)	1 (ref)	1 (ref)	1 (ref)
101–200, mg/month	6448	1.006 (0.915–1.107)	1.088 (0.998–1.186)	1.068 (0.939–1.214)	1.028 (0.957–1.104)
201–300, mg/month	1180	1.093 (0.895–1.335)	1.117 (0.93–1.341)	1.155 (0.882–1.514)	1.114 (0.955–1.3)
301–400, mg/month	284	1.106 (0.787–1.553)	0.926 (0.669–1.283)	0.943 (0.573–1.554)	1.135 (0.874–1.475)
401+, mg/month	79	1.5 (0.818–2.749)	1.222 (0.674–2.215)	1.481 (0.656–3.342)	1.261 (0.75–2.12)

Hazard ratios were adjusted for age, sex, medical aid status, and comorbid conditions.

**Table 4:** Risk of outcomes between high- and low-dose groups after propensity score matching.

	Unadjusted HR (95% CI)	Adjusted HR (95% CI) *
Infection-related hospitalization		
1–299, mg/month	1 (ref)	1 (ref)
≥300, mg/month	1.109 (0.846–1.453)	1.112 (0.847–1.458)
Cardiovascular events		
1–299, mg/month	1 (ref)	1 (ref)
≥300, mg/month	0.971 (0.751–1.255)	0.940 (0.727–1.217)
All-cause mortality		
1–299, mg/month	1 (ref)	1 (ref)
≥300, mg/month	1.086 (0.727–1.623)	1.078 (0.720–1.612)
All-cause hospitalization		
1–299, mg/month	1 (ref)	1 (ref)
≥300, mg/month	1.091 (0.941–1.263)	1.088 (0.938–1.261)

* Adjusted for age, sex, medical aid status, and comorbid conditions.

**Table 5:** Subgroup analysis of the risk of outcomes in the high-dose versus the low-dose group.

	Number at Risk	Infection-Related Hospitalization HR (95% CI)	Cardiovascular Events HR (95% CI)	All-Cause Mortality HR (95% CI)	All-Cause Hospitalization HR (95% CI)
Age					
≥65 years	15,772	1.016 (0.667–1.547)	0.855 (0.577–1.268)	1.167 (0.724–1.881)	1.225 (0.843–1.780)
<65 years	17,755	1.199 (0.828–1.734)	0.958 (0.671–1.369)	0.774 (0.377–1.590)	1.047 (0.801–1.367)
Sex					
Men	20,259	1.366 (0.996–1.874)	0.940 (0.687–1.287)	1.016 (0.645–1.601)	1.159 (0.893–1.506)
Women	13,268	0.604 (0.327–1.113)	0.863 (0.523–1.425)	1.008 (0.454–2.239)	0.996 (0.675–1.471)
Diabetes					
Yes	19,684	1.036 (0.742–1.447)	0.862 (0.630–1.180)	1.061 (0.679–1.657)	1.107 (0.842–1.455)
No	13,843	1.308 (0.792–2.159)	1.044 (0.638–1.709)	0.878 (0.374–2.065)	1.098 (0.769–1.569)
Ischemic heart disease					
Yes	10,760	1.106 (0.702–1.742)	0.793 (0.531–1.186)	1.192 (0.687–2.068)	0.917 (0.631–1.333)
No	22,767	1.107 (0.778–1.575)	1.013 (0.715–1.436)	0.866 (0.488–1.539)	1.210 (0.929–1.576)
Heart failure					
Yes	8388	0.924 (0.545–1.564)	1.011 (0.645–1.586)	0.84 (0.412–1.713)	1.139 (0.739–1.754)
No	25,139	1.204 (0.868–1.67)	0.857 (0.615–1.195)	1.103 (0.687–1.772)	1.094 (0.852–1.406)
Medical aid recipient					
Yes	8214	1.625 (0.988–2.672)	0.891 (0.528–1.504)	1.152 (0.488–2.718)	0.891 (0.579–1.372)
No	25,313	0.960 (0.685–1.347)	0.931 (0.684–1.268)	1.014 (0.650–1.582)	1.194 (0.929–1.535)

Hazard ratios were adjusted for age, sex, medical aid status, and comorbid conditions.

## Data Availability

The data presented in this study are available on request from the corresponding author. The data that support the findings of this study are available from the National Health Insurance Service (NHIS) in South Korea. NHIS data is restricted access to protect the confidentiality of the data and is available upon reasonable request and authorization from the National Health Insurance Administration. Information can be found at https://nhiss.nhis.or.kr/en/z/a/011/lpza011m01en.do.

## Supplementary Materials

The following supporting information can be downloaded at https://www.mdpi.com/article/10.3390/jcm14010063/s1, File S1: ICD-10 codes for identifying events and comorbidities; File S2: Baseline characteristics before & after propensity score matching in high and low-dose groups.
