# Supplementary material for: Safety of High-Dose Intravenous Iron in Hemodialysis Patients: Results from the National Health Insurance Service (2019–2020) in South Korea
Source: J Clin Med. 2024 Dec 26;14(1):63. doi: 10.3390/jcm14010063 (PMC11721372; doi:10.3390/jcm14010063)
Supplement: Supplementary file 1 [file jcm-14-00063-s001.zip › jcm-3362392-supplementary.pdf]

<Supplementary file S1>

ICD-10 and procedure codes for identifying events

Infection-related hospitalization	A00-A09, A30-A49, B95-B97, B99, A15-A19, G00-G09, J00-06, J09-J18, J20-J22, J30-J39, J85-J86, K61, K81, N10, N30, N39.0
Cardiovascular event	I20-25<br>Procedure code: M6551~4 (PTCA), M6561~7 (PCI), M6571~2, M6634 (thrombolytic treatment.), O1640~2 (operation), O1647~9, OA640~2, OA647~9

ICD-10 codes for comorbidities

Diabetes	E10-14
Hypertension	I10-13, I15, R03.0
Heart failure	I11.0, I13.0, I13.2, I13.9, I42, I43, I50
Ischemic heart failure	I20-25
Cerebrovascular disease	I60-69
Chronic obstructive lung disease	J40-47, J98.2, J98.3
Liver cirrhosis and liver failure	K70.2, K70.3, K71.7, K74, K76.6, K76.7, I85.0, I85.9, I98.2, I98.3, K70.4, K72
Cancer	C00-97

<Supplementary file S2>

Supplementary Table. Baseline characteristics before & after propensity score matching in high and low-dose groups

	Before PSM			Post PSM		
	Low-dose iron (n=13,609)	High-dose iron (n=363)	SMD	Low-dose iron (n=1815)	High-dose iron (n=363)	SMD
Age (years)	63.01 ±12.95	59.63 ±13.05		60.25 ± 13.16	59.63 ± 13.05	
18-64	7,249 (53.27)	231 (63.64)	0.227	1,154 (63.58)	231 (63.64)	0.006
≥65	6,360 (46.73)	132 (36.36)	0.227	661 (36.42)	132 (36.36)	0.006
Sex (male)	8,016 (58.90)	252 (69.42)	0.221	1,269 (69.92)	252 (69.42)	0.01
Sex (female)	5,593 (41.10)	111 (30.58)	0.221	546 (30.08)	111 (30.58)	0.01
Medicaid	3,115 (22.89)	90 (24.79)	0.045	479 (26.39)	90 (24.79)	0.038
Comorbid conditions						
Diabetes	8,013(58.88)	235(64.74)	0.12	1,162 (64.02)	235 (64.74)	0.015
Hypertension	10,388(76.33)	298(82.09)	0.141	1,508 (83.09)	298 (82.09)	0.025
Heart failure	3,329(24.46)	97(26.72)	0.052	453 (24.96)	97 (26.72)	0.04
Ischemic Heart Disease	4,505(33.10)	123(33.88)	0.016	585 (32.23)	123 (33.88)	0.035
CVD	1,744(12.82)	42(11.57)	0.036	236 (13.00)	42 (11.57)	0.044
COPD	3,999(29.39)	114(31.41)	0.044	545 (30.03)	114 (31.41)	0.03
Liver cirrhosis and failure	409(3.01)	12(3.31)	0.017	40 (2.20)	12 (3.31)	0.063
Cancer	939(6.90)	34(9.37)	0.091	169 (9.31)	34 (9.37)	0.002

Data are number (percent) and mean (standard deviation)

Abbreviations: PSM, propensity score matching; CVD, cerebrovascular disease; COPD, chronic obstructive lung disease
